# Syndrome de loges compliquant un tennis leg: rapport de cas

**DOI:** 10.11604/pamj.2020.37.310.22444

**Published:** 2020-12-03

**Authors:** Aniss Chagou, Hamza Benameur, Ali Zine, Salim Bouabid, Moustapha Boussougua, Abdeloihab Jaafar

**Affiliations:** 1 Mohammed VI University of Health Sciences (UM6SS), Casablanca, Morocco

**Keywords:** Syndrome de loges, tennis leg, aponévrotomie, Compartment syndrome, tennis leg, aponeurotomy

## Abstract

Le “tennis leg” est une désinsertion d´une partie ou de la totalité du gastrocnémien médial de sa jonction musculo-tendineuse. Bien traitée, cette lésion musculaire évolue généralement favorablement. Le “tennis leg” n´est que très rarement associé à des complications majeures tels que le syndrome de loges aigu. Nous rapportons le cas d´un patient sportif, Judoka victime d´une désinsertion de son gastrocnémien droit compliquée d´un syndrome de loges aigu. Une aponévrotomie chirurgicale en urgence a été nécessaire.

## Abstract (en)

Tennis-leg is a partial or total disinsertion of the medial head of the gastrocnemius muscle from its musculoskeletal junction. Patients receiving adequate treatment generally have favorable outcome. Tennis leg is rarely associated with major complications such as acute compartment syndrome. We report the case of a judo athlete with disinsertion of the right medial head of the gastrocnemius muscle complicated by acute compartment syndrome. Emergency aponeurotomy was required.

## Introduction

Le “tennis leg” est une désinsertion d´une partie ou de la totalité du gastrocnémien médial de sa jonction musculo-tendineuse. Bien traitée, cette lésion musculaire évolue généralement favorablement. L´association d´un “tennis leg” à un syndrome de loges est exceptionnelle. Un diagnostic rapide suivi d´une prise en charge chirurgicale par aponévrotomie sont indispensables pour éviter des complications gravissimes pour le patient aussi bien fonctionnelles que vitales.

## Patient et observation

**Figure 1:**
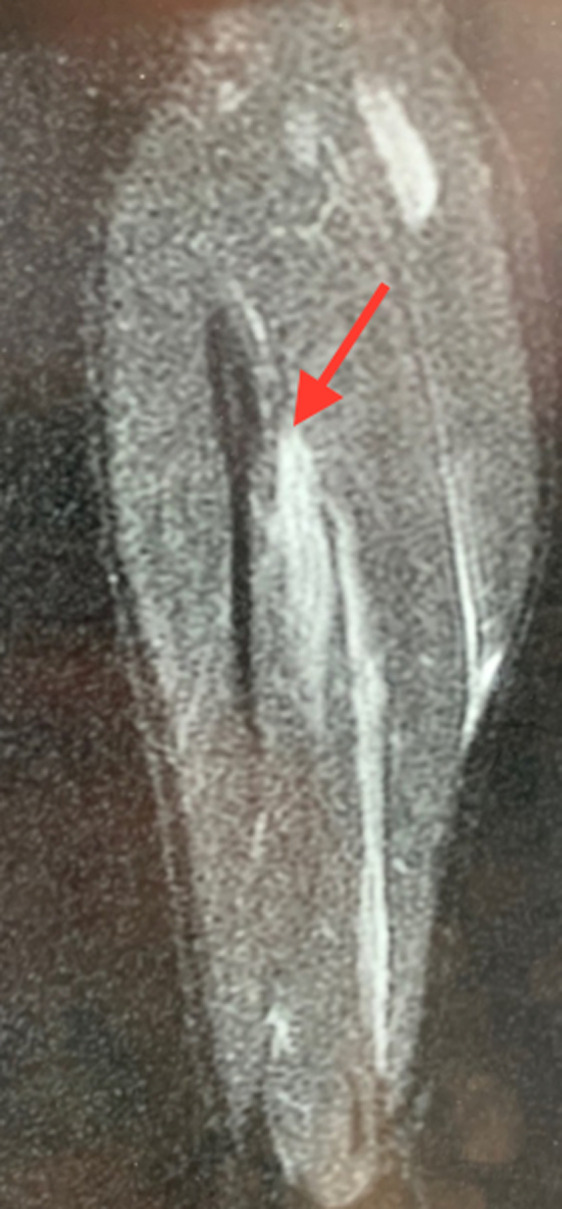
image d´une coupe longitudinale d´une IRM de la jambe montrant une lésion myo-aponévrotique du gastrocnémien médial grade 3

**Figure 2:**
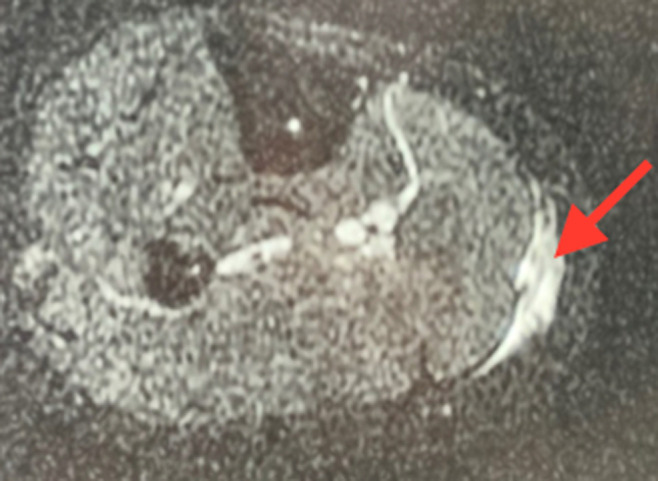
image d´une coupe transversale d´une IRM de la jambe montrant une lésion myo-aponévrotique du gastrocnémien médial grade 3

Nous rapportons le cas d´un patient de 32 ans, judoka participant à des compétitions de niveau régional. Le patient a ressenti durant son combat une douleur aiguë au milieu du mollet. Il a déclaré ressentir une sensation de coup de fouet et entendu un claquement suivis d'un gonflement immédiat et d'une impotence fonctionnelle totale. Le patient se trouvait en position d´impulsion avec le genou en extension et la cheville en flexion dorsale à 90°. Le patient a dû abandonner le combat. Une heure après, le patient a pu remarcher bien que difficilement. Une imagerie par résonance magnétique (IRM) a été réalisée au niveau de la structure locale qui a objectivé une désinsertion partielle du jumeau interne au niveau de sa jonction musculo-tendineuse (Figure 1 et Figure 2). Un traitement antalgique a été prescrit. Le patient a par la suite rejoint le bus avec une durée de trajet de cinq heures.

Nous avons reçu le patient 8 heures après son traumatisme. Il se plaignait de douleurs intenses au niveau la jambe. Aucune ecchymose n'a été trouvée au-dessus de la jambe. Le mollet était sensible à la palpation sur la face postérieure de la jambe gauche, avec une peau tendue dans les compartiments superficiels postérieur, antérieur et latéral. Le pied était maintenu dans une position d'équin antalgique, avec une douleur intense constatée lors de tout mouvement de dorsiflexion mais aucune douleur avec l'étirement du fléchisseur plantaire de l'orteil. Le patient présentait par ailleurs des paresthésies dans le territoire du nerf péronier profond. Les pouls pédieux et tibial postérieur étaient perceptibles.

Nous nous sommes appuyés sur l´IRM initialement réalisée pour éliminer un syndrome de loges sur une fracture ou une rupture d´anévrysme. Le diagnostic retenu est celui de syndrome de loges secondaire à une désinsertion du gastrocnémien médial de la jonction musculo-tendineuse. Le patient a été pris en charge au bloc opératoire une heure après son arrivée. Une incision longitudinale de 20cm était centrée sur le gastrocnémien médial. Après section du fascia profond, un hématome dense a été observé dans la jonction musculo-tendineuse gauche de l´extrémité proximale du muscle gastrocnémien média. L'hématome consistait en environ 100ml de sang coagulé et a été évacué. Le chef médial du muscle gastrocnémien gauche était partiellement rompu à sa jonction musculo-tendineuse. Suite à la décompression du compartiment postérieur superficiel, la palpation a montré que la tension des tissus mous de toute la jambe avait considérablement diminué.

L´incision a été refermée sans greffe de peau au bout de 5 jours et le patient a été immobilisé pendant 3 semaines dans un plâtre cruro-pédieux genou fléchi à 60° et cheville en flexion plantaire à 30°. Le genou a été libéré après 3 semaines. La jambe a été maintenue dans une botte de marche avec des talonnettes permettant une diminution progressive de l´équin. Des séances de rééducation ont été prescrites à base d´étirements manuels progressifs, stretching et réentraînement pliométrique.

## Discussion

Les blessures aiguës du mollet sont courantes aussi bien chez les athlètes que chez les non-athlètes. Parmi ces blessures, les lésions du tendon d´Achille sont de loin les plus fréquentes. Les lésions des autres muscles du mollet restent beaucoup moins fréquentes, elles doivent cependant être prises en compte pour assurer le diagnostic et le traitement appropriés des patients présentant des lésions au mollet. La désinsertion du jumeau interne est classiquement l´apanage du sportif d´âge mûr, aux alentours de 40 ans. Le “tennis leg” résulte le plus souvent d´un mécanisme endogène qui associe une contraction maximale brutale du muscle à un étirement asynchrone du système suro-achilléo-calcanéo-plantaire: impulsion, le genou étant en extension et la cheville en flexion dorsale [1,2]. À l´examen, l´appui est très douloureux, voire impossible du côté blessé et le sujet se déplace précautionneusement sur la pointe du pied, genou fléchi, en esquivant le demi-pas postérieur. Le mollet apparaît tendu, gonflé, œdématié. Une ecchymose survient, dans les jours suivant l´accident, à la face postérieure de la jambe pour s´étendre secondairement vers les gouttières rétro-malléolaires.

Si le “tennis leg” est une pathologie relativement courante, le syndrome de loges secondaire à cette affection est par contre très rare. Très peu de cas rapportent un syndrome de loges sur une désinsertion musculaire ou une rupture musculaire [3-5]. Un seul cas rapportant un syndrome de loge secondaire à un tennis leg a été rapporté dans la littérature [3]. Le délai d´apparition du syndrome de loges peut être différent dans ces cas. Un cas publié portant sur un tennis leg chez un ingénieur de 51 ans après une promenade sans aucun traumatisme, diagnostiqué plus tard comme étant un syndrome de loges aigu [3], impliquait un intervalle entre la lésion et l'apparition des symptômes de 20 jours. Ce patient avait poursuivi ses activités habituelles après l'événement en cause. Les rapports de cas publiés précédemment décrivant un syndrome de loges secondaire à une déchirure musculaire après une blessure mineure ou des événements atraumatiques, relève une période prolongée (typiquement >10 heures) après l'événement ayant provoqué le développement d'un syndrome de loges [5-7].

Le délai d´apparition des symptômes chez notre patient est de 7 heures; le patient ayant pris le bus avec un trajet de cinq heures. La jambe a été maintenue pendante durant ce trajet ce qui a aggravé l'hémorragie dans le compartiment postérieur superficiel et augmenté la pression du compartiment. Le délai de prise en charge de ces syndromes de loges est extrêmement important. Tout retard de diagnostic peut impliquer des complications gravissimes pour le patient aussi bien fonctionnelles que vitales. Une fasciotomie urgente avec évacuation de l´hématome est le seul traitement capable d´éviter ces complications [5].

Les signes cliniques sont d´une aide précieuse au diagnostic, la douleur est profonde, constante, souvent mal localisée, disproportionnée par rapport aux résultats physiques; souvent peu sensible aux analgésiques. La douleur est accentuée par l'étirement passif du compartiment impliqué. Les compartiments affectés sont fermes et non compressibles. Le patient ressent un engourdissement ou «picotements» dans la distribution cutanée des nerfs traversant le compartiment affecté. A l´examen physique le membre est pâle, mais l'extrémité peut aussi paraître tachée. La paralysie musculaire peut être liée à la douleur. Quand la vraie paralysie est présente, c'est un mauvais pronostic pour la récupération [8]. La mesure de la pression intra-compartimentale peut être utile pour confirmer le diagnostic. Si elle doit être réalisée dans les trois compartiments pour éviter les faux négatifs.

## Conclusion

Le “tennis leg” peut provoquer un syndrome des loges des compartiments antérieur, latéral et postérieur. Cela a été rapporté par Straehley et Jones [9] et Pai et Pai [3]. Dans le cas rapporté par Pai et Pai [3], la décompression du compartiment postérieur seul a diminué la pression dans les compartiments antérieur et latéral et évité une fasciotomie inutile du compartiment antérieur. Nous avons réalisé une fasciotomie postérieure en supposant que la décompression de la loge postérieure permettrait de baisser la pression des loges latérale et antérieure.
